# Discovery of crystalline Fe_2_O_3_ in returned lunar soils

**DOI:** 10.1126/sciadv.ady5169

**Published:** 2025-11-14

**Authors:** Yiheng Liu, Haijun Cao, Rui Li, Jian Chen, Chengxiang Yin, Ziyi Jia, Xuejin Lu, Le Qiao, Xiaohui Fu, Changqing Liu, Chen Li, Yanqing Xin, Ying-bo Lu, Xiaojia Zeng, Jianzhong Liu, Yang Li, Zongcheng Ling

**Affiliations:** ^1^ Shandong Provincial Key Laboratory of Optical Astronomy and Solar-terrestrial Environment, School of Space Science and Technology, Institute of Space Sciences, Shandong University, Weihai 264209, P.R. China.; ^2^ Center for Lunar and Planetary Sciences, Institute of Geochemistry, Chinese Academy of Sciences, Guiyang 550081, P.R. China.; ^3^ School of Engineering, Yunnan University, Kunming 650091, P.R. China.

## Abstract

Lunar materials were believed to have formed and been preserved in a reducing environment, with only Fe^2+^ and Fe^0^ present. Native oxidized minerals, such as hematite, have not been validated in previously returned lunar samples. In this work, we report the discovery of micrometer-scale crystalline Fe_2_O_3_ in the forms of hematite (α-Fe_2_O_3_) and maghemite (γ-Fe_2_O_3_) overgrowing troilite in the recently returned Chang’e-6 (CE6) lunar soils. It is possible that impact-induced oxygen release created localized micrometer-scale regions of elevated oxygen fugacity, facilitating the formation of Fe_2_O_3_ at temperatures between ~700° and 1000°C. This finding provides credible evidence for the presence of Fe_2_O_3_ on the lunar surface, challenging the traditional understanding of lunar surface redox states. In addition, the Fe_2_O_3_ in the form of maghemite may be the mineralogical reason for the generation of the magnetic anomalies observed around the South Pole–Aitken basin.

## Teaser

The discovery of large Fe_2_O_3_ crystals in Chang’e-6 soils provides evidence of oxidizing environments on the lunar surface.

## INTRODUCTION

The giant-impact hypothesis of lunar formation suggests a compositional similarity to Earth’s mantle (*1*, *2*). Previous works have demonstrated that the oxygen fugacity of the lunar material was notably lower than that of the Earth (*3*), and the giant impact origin should have led to a loss of volatiles, including oxygen, in the primitive Moon (*4*). The prolonged low oxygen fugacity of lunar magmatic processes (*3*, *5*), coupled with continuous solar wind irradiation (*6*–*8*), has further promoted reduced valence states of elements within lunar minerals. In the past decades, lunar materials were believed to have formed and been preserved in a reducing environment (*9*, *10*). Until recently, no mineralogical evidence of hematite (α-Fe_2_O_3_) has been confirmed from returned lunar samples or remote sensing observations. However, ferric iron–bearing materials, including magnetite and iron hydroxides, have been identified ambiguously in returned Apollo sample specimens (*11*–*14*). Nevertheless, experimental studies have shown that ferric iron–bearing minerals such as akaganéite, goethite, and lepidocrocite are unstable on the lunar surface, indicating that the iron hydroxides in Apollo samples likely resulted from terrestrial contamination (*14*). Remote sensing observations by the Moon Minerology Mapper have indicated the widespread presence of highly oxidized hematite (α-Fe_2_O_3_) at high latitudes (above 75°) (*15*). A hypothesis has been proposed that oxygen delivered from the Earth’s upper atmosphere could act as the primary oxidant forming lunar hematite. However, this remote observation of hematite requires further exploration by future landing and sampling missions. Furthermore, a recent work has suggested that these observations may be false signatures in the orbital spectroscopic datasets (*16*). In addition, a trace amount of nanophase magnetite has been identified in Chang’e-5 (CE5) lunar soils by using state-of-the-art high-resolution microscopic analysis techniques (*17*, *18*). Despite these findings, compelling mineralogical evidence that hematite exists on the lunar surface has remained insufficient.

In this work, we report the discovery of micrometer-scale crystalline Fe_2_O_3_ in Chang’e-6 (CE6) lunar samples and then characterize its occurrence and mineralogy (hematite and maghemite). We hypothesize a reasonable conjecture that the formation of Fe_2_O_3_ is driven predominantly by vapor-phase deposition following thermal oxidation during large-scale impact events, not space weathering processes. This process may contribute to the magnetization of materials surrounding the South Pole–Aitken (SPA) basin, which plays a substantial role in the formation of lunar farside magnetic anomalies.

## RESULTS

### Identification of crystalline hematite and maghemite

**Fig. 1.**
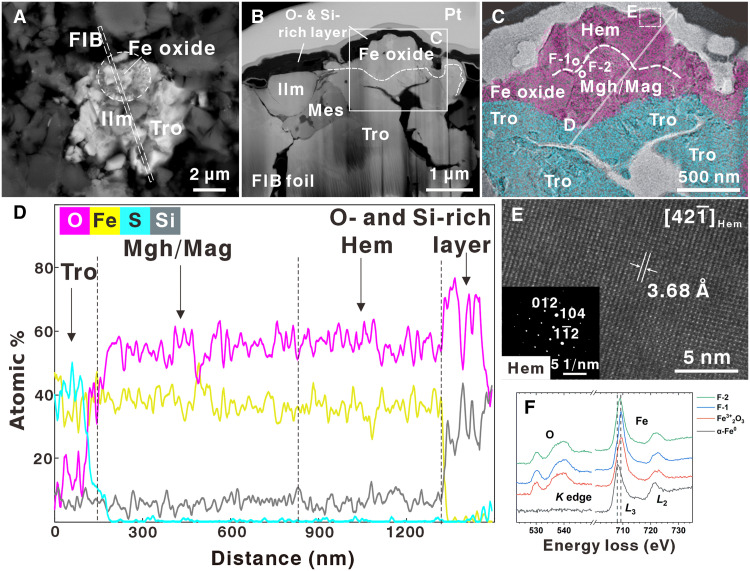
Morphology, composition, and crystal structure of the studied iron oxide mineral in the CE6 breccia clast sample (CE6C0300YJFM001GP003). (**A**) BSE image of the Fe_2_O_3_-bearing clast in the polished section. (**B**) BSE image of the FIB foil, which was taken along the dashed rectangle in (A). (**C**) Distinctive boundary between iron oxide (magenta) and troilite (cyan) grain, as identified by elemental maps. The dashed lines represent the boundaries between hematite and maghemite/magnetite. (**D**) Quantitative TEM-EDX profiles along the white arrow in (C). (**E**) High-resolution TEM image of a hematite (α-Fe_2_O_3_) grain. The fast Fourier transform (FFT) pattern is shown in the bottom-left corner. (**F**) Fe *L*_2,3_ electron energy-loss spectra of Fe^3+^-bearing grains and comparison with standard data (Fe^3+^ and Fe^0^). Hem, hematite; Mgh, maghemite; Mag, magnetite; Tro, troilite; Ilm, ilmenite; Mes, mesostasis.

Nine hematite (α-Fe_2_O_3_)–bearing grains, associated with ferrous iron minerals such as troilite and ilmenite (Fig. 1A), were initially identified by Raman spectroscopic analyses of CE6 soils (figs. S1 and S2). A fragment containing micrometer-scale hematite (~3 μm by 1.7 μm) in breccia clast (CE6C0300YJFM001GP003; fig. S3) was selected for further measurements. The hematite was observed on the surface of a troilite grain in backscattered electron (BSE) imagery and energy-dispersive spectroscopy (EDS) element maps of the breccia clast (Fig. 1A and fig. S4). A focused ion beam (FIB) foil was extracted from this fragment to conduct additional microscopic validation (Fig. 1A). The hematite in the FIB foil was found to be a hat-shaped Fe_2_O_3_ grain lying on top of troilite (Fig. 1B). The brim of the hat-shaped Fe_2_O_3_ grain extends with a thickness ranging from 100 to 300 nm. Subsequently, two Fe_2_O_3_ phases (hematite and maghemite) were identified by combined chemical and microstructural analyses of the FIB foil (Fig. 1).

Quantitative transmission electron microscopy (TEM)–energy-dispersive x-ray (EDX) compositional maps revealed that the hematite grain is coated by a thin layer rich in Si and O (Fig. 1B), indicating that the Fe_2_O_3_ was not generated by laser heating. This Si- and O-rich layer was not observed in Raman spectroscopic analyses because of its featureless signature. The absence of discernible Raman peak features indicates that the layer is highly amorphous, lacking any structural remnants of crystalline precursors and consistent with a fully glassy, noncrystalline phase. TEM observations revealed a homogeneous thickness and an absence of flow textures or morphologies typically associated with melt ejecta. EDX compositional measurements showed that the layer consists exclusively of Si and O, with no detectable concentrations of major rock-forming elements (e.g., Al, Fe, Mg, and Ca). These observations suggest that this layer is most plausibly a glassy condensate formed from a Si- and O-rich vapor plume (*19*). Alternative origins, such as part of a quenched melt, cannot be fully excluded given the small size of the grain studied.

The boundary between iron oxides and troilite is clearly delineated in the EDX compositional maps (Fig. 1C). The troilite exhibits intragrain microcracks, whereas the oxide grains remain free of fractures (Fig. 1). This microstructure indicates that the troilite grain might have been sourced from the fragmentation of the basaltic parent rock and that the oxide grains were derived from postfragmentation processes. However, there is no evidence indicating that the troilite grain did not undergo one or more recyclings through multiple breccia-forming events. Elemental mapping further confirmed that the ferric iron–bearing grain consists exclusively of Fe and O, with no other detectable elements (table S1). The Fe/O atom ratio, ranging from 0.62 to 0.70, is consistent with a Fe_2_O_3_ composition (Fig. 1D and table S1).

The high-angle annular dark-field (HAADF) images confirmed that the iron oxide portion is well crystallized and consists of multiple submicrometer monocrystals with distinct boundaries (fig. S5). This suggests that the hematite formation may have involved the development of multiple crystal nuclei. High-resolution TEM images of the upper portion of the iron oxide particle along the $\left[42\bar{1}\right]$ zone axis indicated (012) lattice fringes with a periodicity of 3.68 Å (Fig. 1E). The angle between (012) and (104) in the selected-area electron diffraction pattern was measured to be 47° (Fig. 1E). These parameters are consistent with the crystal structure of hematite (α-Fe_2_O_3_). Moreover, the electron energy-loss spectroscopy (EELS) iron *L*_2,3_ edge at ~708.0 eV was the clearest diagnostic features for identifying Fe^3+^ (Fig. 1F, red line). These in situ microscopic evidences were consistent with the Raman results.

**Fig. 2.**
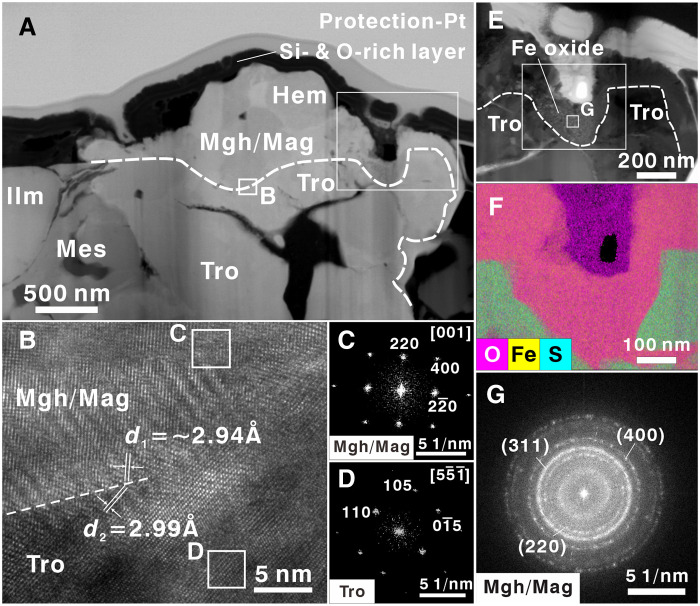
Identification of mineral assemblage around hematite in the CE6 soil fragment. (**A**) BSE image of the FIB foil with labels of mineral phases. Hem, hematite; Mgh, maghemite; Mag, magnetite; Tro, troilite; Ilm, ilmenite; Mes, mesostasis. (**B**) High-resolution TEM image of the contact surface between magnetite and troilite in the white box in (A). (**C** and **D**) FFT patterns of magnetite and troilite, respectively, in (B). (**E**) Bright-field TEM image of a typical O-rich area. (**F**) TEM-EDX map results from the white box in (E). (**G**) FFT pattern of the polycrystalline diffraction of maghemite/magnetite.

Further structural characterization confirmed the presence of iron oxides (Fig. 2A). TEM results indicated that, despite no obvious difference in chemical composition of iron oxides, the interplanar spacing of the mineral grain between hematite and troilite was 2.94 to 2.96 Å, corresponding to the (220) lattice planes. The iron *L*_2,3_ spectrum showed a characteristic peak at ~709.0 eV, positioned between the reference spectra of Fe^2+^ (708.0 eV) and Fe^3+^ (709.5 eV), indicating the coexistence of both valence states in the intermediate layer, i.e., dominance of magnetite (Fe_3_O_4_). Because of the similarities of lattice structure and cell parameters, maghemite (γ-Fe_2_O_3_) likely exhibits a paragenetic relationship with magnetite at its contact with the hematite (α-Fe_2_O_3_). High-resolution TEM imaging also clearly demonstrated a paragenetic relationship at the iron oxide–troilite interface (Fig. 2, B to D).

## DISCUSSION

### Potential formation mechanism of Fe_2_O_3_

Previous studies have proposed several formation mechanisms of ferric iron on the Moon, including both endogenous (*20*–*24*) and exogenous (*25*) mechanisms. In this work, the Fe^3+^-bearing minerals observed in the CE6 samples occurred primarily within lunar regolith breccia, rather than in igneous basalt fragments. This observation suggests that oxidization did not occur in the volcanic events.

An alternative possibility is that the hematite particle may have been oxidized by oxygen from the Earth’s upper atmosphere (*15*), derived through a phenomenon commonly referred to as Earth wind (*26*). In this scenario, reactive oxygen species (e.g., oxygen ions) in the Earth wind serve as an oxidant, which facilitates the spontaneous oxidation of ferrous iron in lunar surface materials. This mechanism is consistent with the prolonged formation of hematite observed in the CE6 sample. However, considering that the penetration depth of Earth wind is typically less than 100 nm (*27*–*29*), the formation of micrometer-scale crystalline hematite cannot be attributed to oxygen implantation by Earth wind. More prominently, the surface of the Fe_2_O_3_ particle observed in this work is coated by a Si- and O-rich glassy layer, which serves as a protective barrier (*30*) against both reducing solar wind and any potential interaction with Earth wind. This glassy layer also provides solid evidence of a vapor deposition mechanism for the Fe_2_O_3_ formation.

**Fig. 3.**
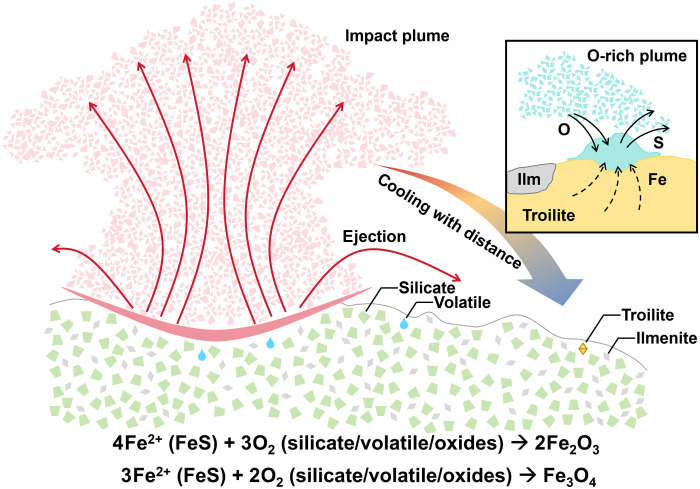
Graphical depiction of the formation scenario of ferric oxides in the CE6 lunar sample.

The Fe^3+^-bearing oxides in the CE6 regolith breccia are likely products of large impact events. During the large impact events, Fe^2+^ ions would have been oxidized to Fe^3+^ under conditions of high temperature and elevated oxygen fugacity. Building on previous studies of lunar Fe^3+^-bearing oxide [e.g., magnetite (*18*)] formation mechanisms, we propose that a shock-heating process during impact events triggered the formation of Fe^3+^-bearing oxidized phases through the release of oxygen during the thermal decomposition of oxygen-rich phases (Fig. 3). During large impact events, oxide and silicate minerals are prone to decompose, releasing oxygen into the surrounding environment. This oxygen release produces a heterogeneous vapor environment characterized by locally high oxygen fugacity, where Fe^2+^ ions are oxidized to higher valence states and ultimately form ferric iron oxides such as hematite and maghemite. This hypothesis was further supported by TEM observations of microcrystalline structures, which suggest a growth relationship between the iron oxide and the underlying troilite (Fig. 2B). This suggests that the ferric iron minerals in CE6 samples were locally oxidized and deposited in a vapor environment.

We propose that this hematite grain was not a product of space weathering but was rather generated by large-scale impact events (e.g., SPA and Apollo) with a high oxygen fugacity plume, which led to the desulfurization of FeS, oxidation of Fe^2+^, and production of Fe_2_O_3_. The Fe_2_O_3_ on the surface of troilite observed in the CE6 samples and the absence of S-rich phases deposited elsewhere suggest that sulfur was likely lost as a volatile species during impact devolatilization. According to the Fe─O─S phase diagram (fig. S6), troilite (FeS) would experience progressive oxidation to magnetite (Fe_3_O_4_) and eventually hematite (Fe_2_O_3_) with increasing oxygen fugacity across a wide temperature range. Comparative phase stability analyses indicate that the transformation from magnetite to hematite (600 to 2000 K) becomes increasingly suppressed at elevated temperatures (fig. S6), suggesting that oxidation of desulfurized troilite is more favorable under relatively low-temperature conditions. Experimental constraints on the troilite desulfurization reaction (*31*), considering the reaction condition of troilite desulfurization, suggest a minimum temperature for Fe vaporization of ~973 K (~700°C) (*29*). This temperature condition lies below the decomposition threshold temperature of ilmenite (~1000°C) (*32*), which is consistent with the presence of ilmenite identified adjacent to the hematite particle. Given that the CE6 landing site is located near the margin of the impact basin, we infer that the observed oxidation occurred within a relatively distal and cooler sector of the ejecta plume with temperatures lower than ~1000°C. Although insufficient for decomposition of ilmenite, such a regional thermal disturbance could still facilitate the desulfurization of troilite, vapor-phase transport, and deposition of iron-bearing phases under oxidizing conditions. This is consistent with the co-occurrence of ilmenite adjacent to the hematite grain, implying that oxidation reactions occurred between 973 and 1273 K (700° to 1000°C). Further support for this interpretation comes from compositional analysis of the glassy layer coating the hematite grain. This amorphous material consists exclusively of Si and O, as revealed by EDX spectroscopy, and lacks other major rock-forming cations. Given that SiO_2_ exhibits the highest saturated vapor pressure among common lunar oxides under impact-related conditions (*33*), we interpret this glassy coating as a vapor-deposited condensate formed near the edge of a large-scale impact plume. However, the size of the impactor remains an open question that requires further modeling and observational evidence.

### Preservation of Fe_2_O_3_ on the Moon

**Fig. 4.**
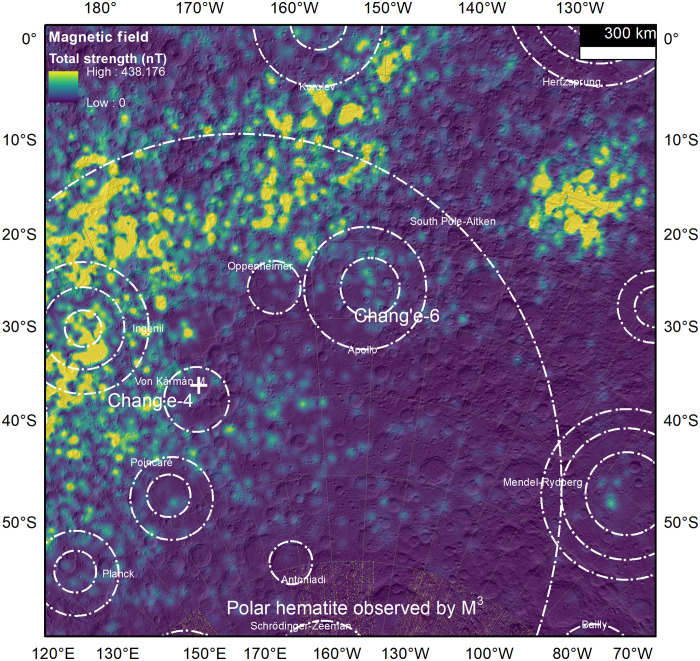
Geological context of the CE6 landing site. The CE6 landing site in the SPA basin is surrounded by the strongest remnant magnetic field on the lunar surface and close to the lunar south polar region where hematite spectral signals were observed. The white dashed circles are basin rings from (*45*).

According to the aforementioned formation hypothesis, micrometer-scale ferric iron oxide minerals should be widely distributed on the lunar surface. However, analyses of previously returned lunar samples (Apollo, Luna, and CE5 missions) have not confirmed any native lunar Fe_2_O_3_ phase. Two possible explanations can account for this discrepancy: the low production rate and rapid exhausting of ferric iron on the lunar surface. First, we attribute the formation of micrometer-scale Fe_2_O_3_ to large-scale impact events. Most previous lunar sampling sites (e.g., Apollo 11, 12, 15, and 17 and CE5) were sites located on the nearside with vast mare coverage, which are less likely to preserve materials from ancient large impact events. As a contrast, the CE6 landing site is located within the SPA basin on the lunar farside (Fig. 4), an area that has undergone numerous large impact events but few lava-filling processes, making it more likely to contain such deposits. In addition, continuous space weathering could lead to the decomposition and reduction of ferric iron into low-valance species, such as Fe^2+^ and Fe^0^ (*34*, *35*). In the absence of a substantial atmosphere, the lunar surface materials are directly exposed to the solar wind, primarily composed of highly reducing hydrogen (*27*). This exposure facilitates the reduction of Fe^3+^ ions, thereby contributing to a reduction in the overall concentration of Fe^3+^-bearing oxides on the lunar surface over time. Thus, regions with weaker solar wind flux are more conducive to the preservation of Fe_2_O_3_. Previous studies have shown that solar wind flux on the Moon can be modulated by factors such as latitude (*35*), magnetic anomalies (*36*, *37*), and topography (*34*, *38*). The solar wind flux decreases as latitude increases (*35*). Because of latitude effects, the CE6 landing site, at the latitude of ~42°S (*39*), experiences a solar wind flux that is significantly lower than the flux at the Apollo and Luna sampling sites. This low flux in the CE6 landing region would benefit the preservation of ferric iron oxides. The presence of magnetic anomalies also plays a significant role in modulating solar wind flux. In the geological context of the CE6 landing region, the strongest remnant magnetic field on the lunar surface is located on the northern rim of the SPA basin (Fig. 4) (*40*–*42*). These magnetic anomalies would reduce the solar wind flux in the CE6 zone (*36*, *37*), as was also observed in the Chang’e-4 landing zone (*43*). The magnetic anomalies provide a protective shielding effect and contribute to the preservation of ferric iron materials in the SPA basin. In addition, the topography of the CE6 landing region may further modulate solar wind flux due to variations in the Mach number (*34*, *38*). The CE6 landing region in the complex ring structures of the SPA and Apollo basins (Fig. 4) may create a localized region of lower solar wind flux. Under these conditions, the regolith at the CE6 landing region likely experienced a relatively minimal solar wind–induced reduction of Fe^3+^, thereby facilitating the preservation of Fe^3+^-bearing materials.

In summary, micrometer-scale crystalline Fe_2_O_3_ was discovered in the CE6 lunar farside sample. We propose that the mechanism responsible for its formation involves impact-induced substantial oxygen release, which oxidizes troilite into hematite and maghemite. The discovery of hematite in the CE6 lunar soil suggests that the upper brecciated zone of the Moon may have undergone more extensive oxidation and produced a greater quantity of oxidizing minerals than previously recognized. In contrast, the micrometer-scale maghemite (γ-Fe_2_O_3_), a typical magnetic mineral, that was identified could also serve as a potential carrier for the magnetic anomalies surrounding the SPA basin on the lunar farside (Fig. 4).

## MATERIALS AND METHODS

### CE6 lunar soil

We obtained lunar surface soil (CE6C0300YJFM001, a total of 3 g) sampled by a mechanical scoop from the China National Space Administration. A rock clast (named CE6C0300YJFM001GP003) was subsequently characterized by various in situ analytical techniques, including Raman spectroscopy, scanning electron microscopy (SEM), EELS, and TEM.

### Sample characterization

Nine micrometer-scale ferric iron–bearing grains were initially identified within the fine-grained CE6 soil sample CE6C0300YJFM001, in which they predominantly coexist with Fe sulfide (troilite) and Ti-Fe oxide (ilmenite). Hematite grains were first recognized on the basis of their characteristic Raman scattering feature at 225.5 and 292.6 cm^−1^, as well as a broadened peak at 1317.9 cm^−1^ (fig. S2). To confirm the presence of ferric iron–bearing phases further, a regolith breccia clast (CE6C0300YJFM001GP003) was selected for preparation of a polished thick section. The largest ferric iron–bearing grain in the CE6 breccia section was then targeted, and an FIB slice was prepared for subsequent TEM examination (Fig. 1A). The FIB profile was selected to cross as many mineral phases as possible, rather than along the crystalline orientation.

### Micro-Raman spectral measurements

Microscopic and Raman spectroscopic analyses of CE6 lunar soils were performed with a Renishaw inVia confocal Raman microscope at Shandong University, Weihai. The excitation laser had a wavelength of 532 nm and a power of ~89 mW, with the laser power adjusted between 0.89 and 8.9 mW. Raman spectra of typical minerals were acquired with a 50-s exposure time, averaged over four accumulations. The spectral range was 70 to 4000 cm^−1^ with a resolution of better than 1 cm^−1^. Using a 50xL long-working distance objective (numerical aperture = 0.5), the laser was focused to a spot of ~2.5 μm. Calibration was performed using a standard Ne lamp and a Si wafer (peak: 520.7 cm^−1^), ensuring Raman shift accuracy within ±0.2 cm^−1^.

### SEM analysis

BSE imagery and elemental x-ray mapping were conducted using an FEI Nano Nova 450 FEG scanning electron microscope equipped with an Oxford X-Max50 EDS at Shandong University, Weihai. The conditions were as follows: 15-kV accelerating voltage, 20-nA beam current, and 7-mm working distance. EDS x-ray elemental mapping was collected from ferric iron minerals within CE6C0300YJFM001GP003, with a dwell time of 120 s. Subsequently, we obtained a Pt-coated ferric iron grain slice using the FEI Scios dual-beam FIB/scanning electron microscope at the Institute of Geochemistry, Chinese Academy of Sciences (CAS). The slice was cut with Ga ions at 30 kV and a beam current of 3 to 15 nA and then embedded in a TEM copper grid.

### EELS and TEM observation

Nanometer-scale structure observations of ferric iron oxide grains were conducted using a Hitachi HF5000 aberration-corrected scanning transmission electron microscope equipped with a Gatan GIF Quantum ER System Model 965 parallel EELS spectrometer at the Shanghai Institute of Ceramics, CAS. Bright-field TEM observations, HAADF scanning TEM, and EDX elemental analyses were conducted with an accelerating voltage of 200 kV and a beam current of 1 to 2 nA. The FIB slice was examined using field-emission TEM (200 kV, FEI Talos F200X) to determine the different mineral phase morphologies in the sample. The chemical composition and crystal structure were acquired using TEM-EDS and fast Fourier transform patterns. EELS was used to confirm the oxidation states of Fe in hematite, maghemite, and magnetite. The EELS detection was performed using a Thermo Fisher Spectra 300 instrument operating at 300 kV. The EELS spectra were obtained using a Dual-EELS instrument with a probe current of 20 pA and a Gatan 1065 K3 EELS receiver. The full width at half maximum of the calibrated zero-loss peak was 0.71 eV, allowing the attainment of high energy resolution.

### Thermodynamic calculation

The thermodynamic data were analyzed in FactSage 8.0 thermodynamic software (Beijing FactTech Ltd., Beijing, China). The reaction module of the FactSage 8.0 software was used to calculate the Gibbs free energy criteria for oxygen involving troilite desulfurization reactions at different temperatures under a low-pressure condition. The equilibrium composition and product formation order of reactions involving oxygen and the different iron ions were calculated using the Equilib module. The accurate application scenario of the Gibbs free energy criterion is a thermodynamic steady-state environment with constant temperature, constant pressure, and no volume work. In this study, the scenario is applicable to thermochemical reactions in a vacuum and at different temperatures. Ion reaction in the impact-generated plume is a nonequilibrium process, but the chemical reaction conforms to the thermodynamic calculation results.
