# Supplementary material for: Discovery of crystalline Fe2O3 in returned lunar soils
Source: Sci Adv. 2025 Nov 14;11(46):eady5169. doi: 10.1126/sciadv.ady5169 (PMC12617526; doi:10.1126/sciadv.ady5169)
Supplement: Supplementary file 1 — Figs. S1 to S6 Table S1 Legend for data S1 [file sciadv.ady5169_sm.pdf]

Science Advances
AAAS

Supplementary Materials for

Discovery of crystalline Fe_2O_3 in returned lunar soils

Yiheng Liu *et al.*

Corresponding author: Zongcheng Ling, zcling@sdu.edu.cn; Yang Li, liyang@mail.gyig.ac.cn; Jian Chen, merchenj@outlook.com

Sci. Adv. **11**, eady5169 (2025)
DOI: 10.1126/sciadv.ady5169

The PDF file includes:

Figs. S1 to S6
Table S1
Legend for data S1

Other Supplementary Material for this manuscript includes the following:

Data S1

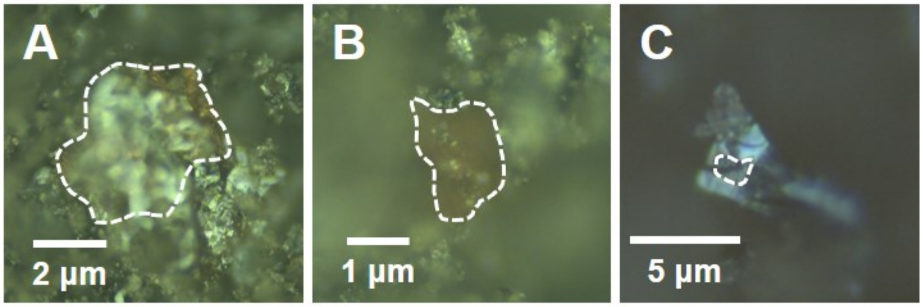

Fig. S1.

Microscopic images of hematite-bearing fragments in CE6 regolith sample. Hematite particles in the regolith sample (dashed polygons) can be identified by their brownish color and occur with ferrous iron-bearing minerals such as troilite and ilmenite. These occurrences are consistent with the ferric iron oxide-bearing fragments observed in the polished section.

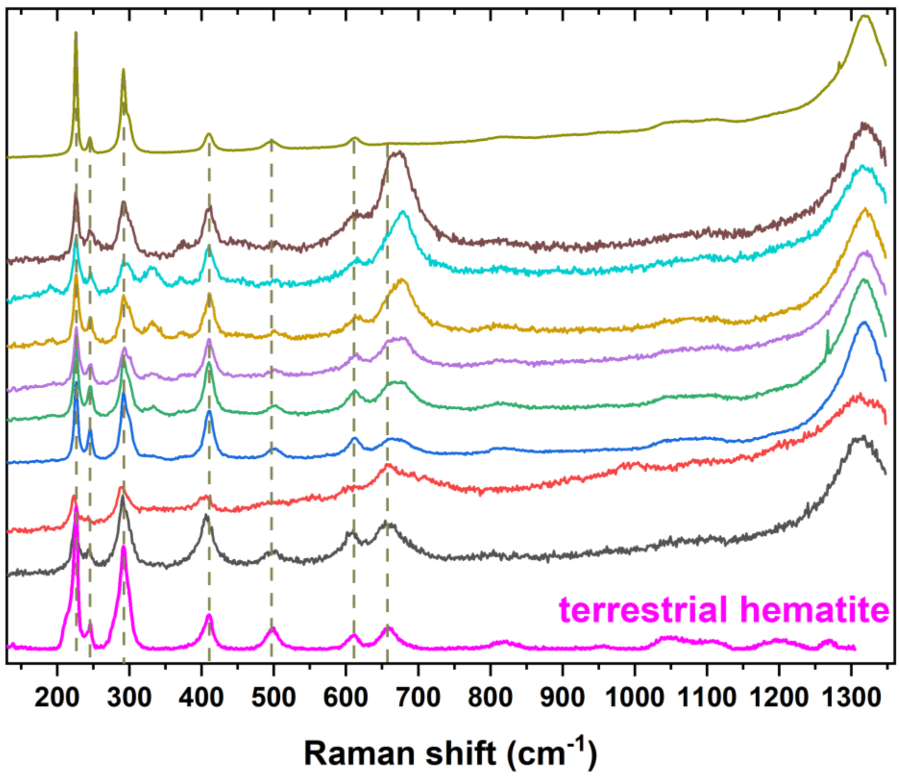

Fig. S2.

Raman spectra of hematite particles identified in CE6 regolith samples. Diagnostic Raman spectral features of hematite are marked by dashed lines. Each spectrum was normalized in intensity. Raman spectral features at around 680 cm^{-1} result from the presence of ilmenite. A Raman spectrum of terrestrial hematite from the RRUFF database (ID: R110013) is shown for comparison.

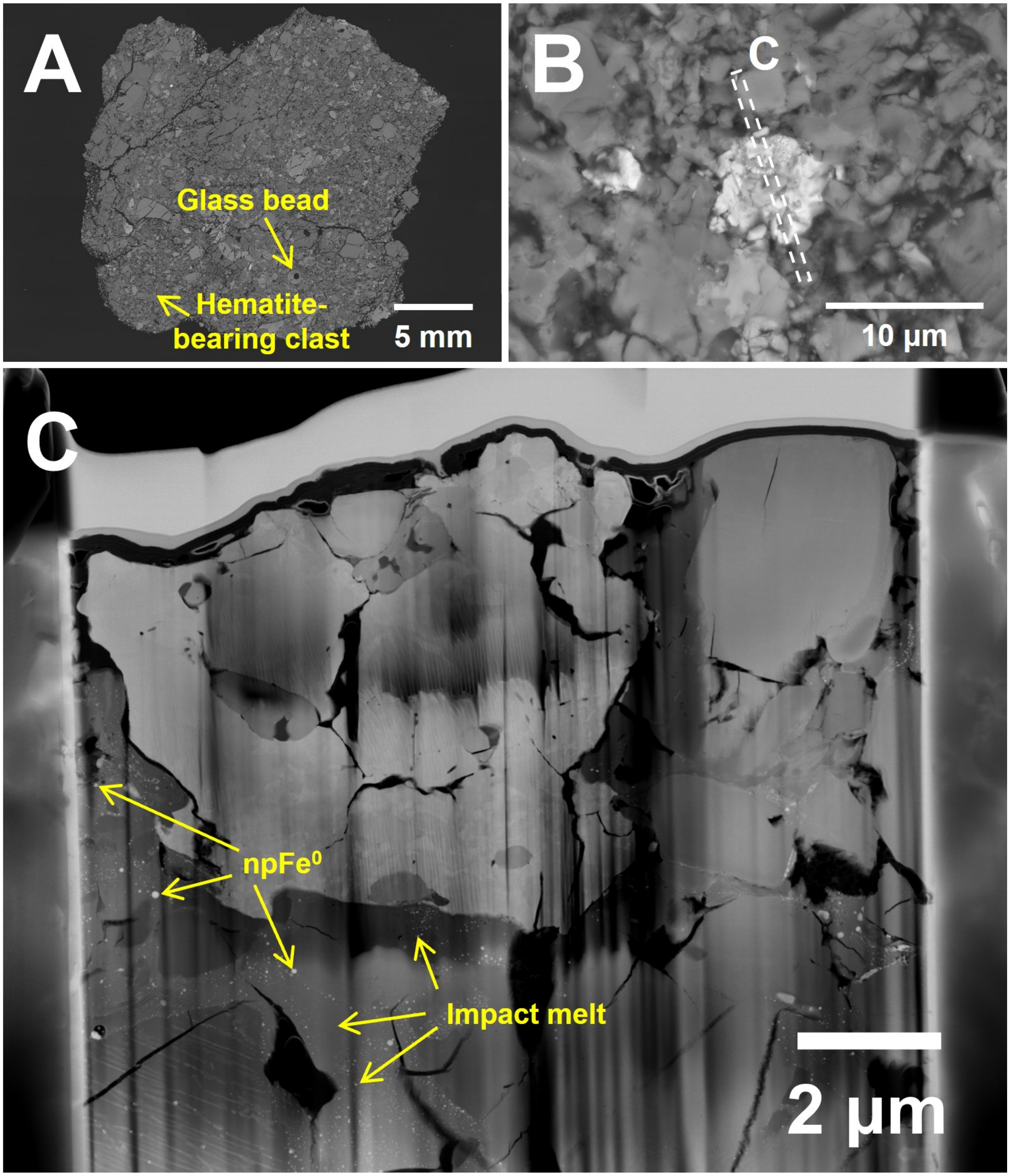

Fig. S3.

Context of the hematite in the CE6 breccia sample CE6C0300YJFM001GP003. (A) BSE image of the whole breccia section. The yellow arrow indicates the Fe_2O_3-bearing grain selected for further measurements. (B) A larger area surrounding the target grain showing its occurrence in the matrix of mineral fragments and lithic clasts. (C) BSE image of the FIB slice showing many Fe^0 globules beneath the polished surface.

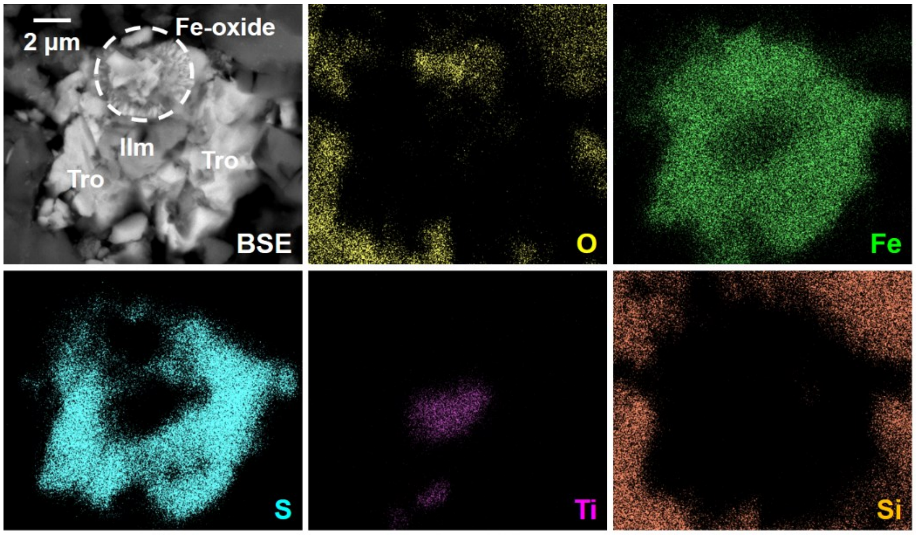

Fig. S4.

Elemental X-ray mapping of hematite-bearing fragment acquired by EDS.

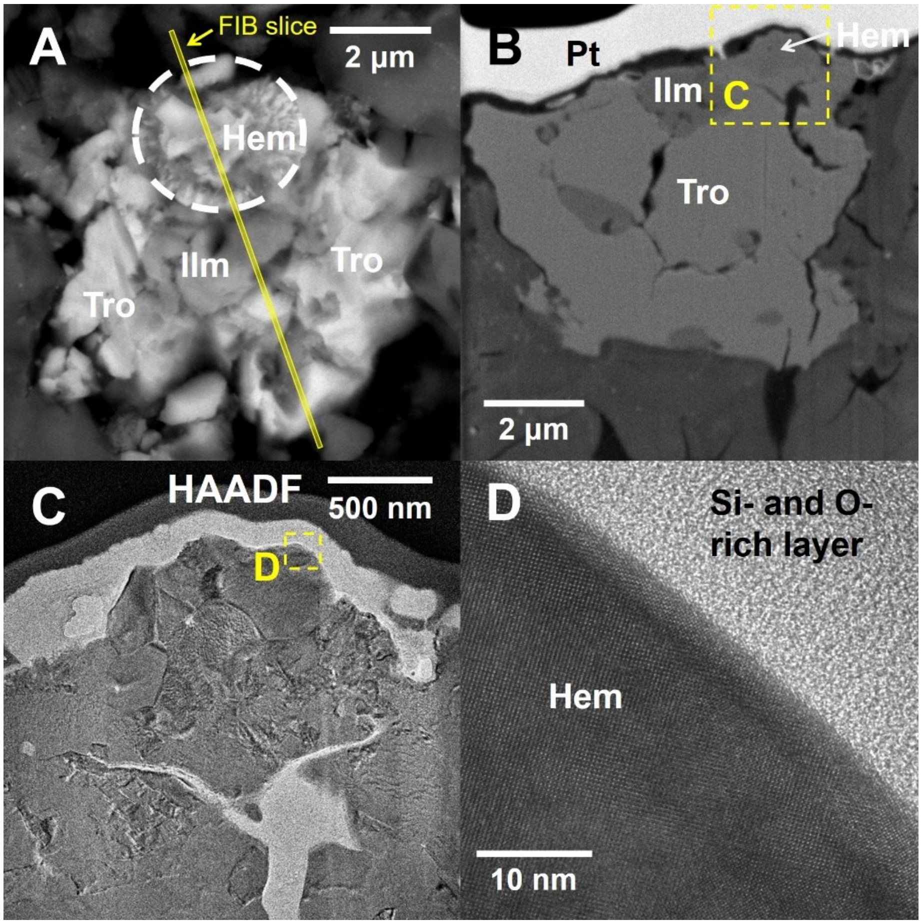

Fig. S5.

TEM results of the ferric iron-bearing mineral assemblages in CE6 breccia CE6C0300YJFM001GP003. (**A**) BSE image of the hematite (Hem) associated with troilite (Tro) and ilmenite (Ilm). (**B**) BSE image of hematite-bearing mineral assemblage in the FIB slice. (**C**) HAADF image of the yellow dashed rectangular region in (**B**). (**D**) HRTEM image of contact surface (the yellow dashed rectangular region in (**C**)) between Fe_2O_3 grain and Si- and O-rich layer.

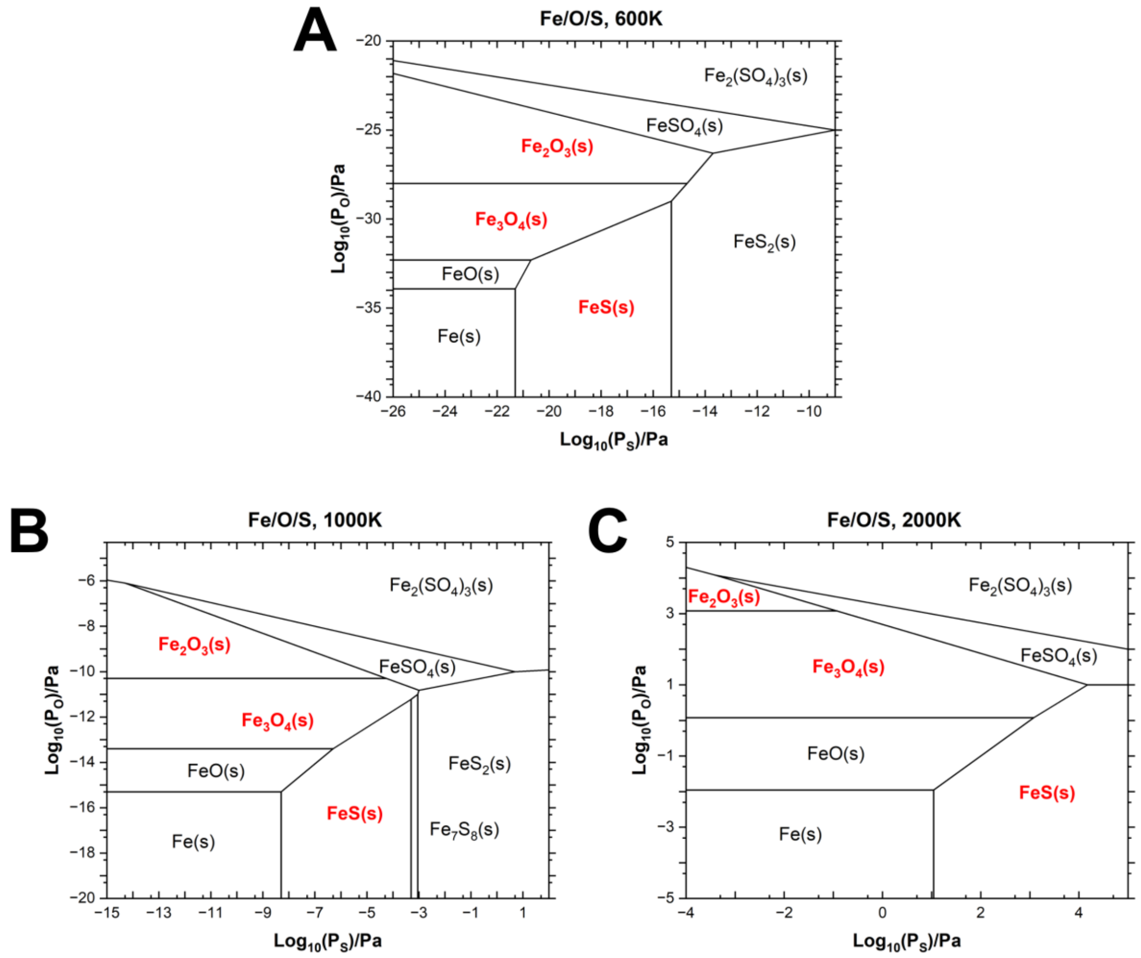

Fig. S6.

Phase diagram of Fe/O/S under different temperature conditions. (A) 600 K, (B) 1000 K, and (C) 2000 K. The thermodynamic calculations demonstrate that the troilite (FeS) phase tends to transit into magnetite (Fe_3O_4) phase and eventually hematite (Fe_2O_3) phase with increase of oxygen fugacity. On the other hand, as the reaction temperature becomes higher, it becomes more difficult for the magnetite phase to transit into hematite phase, which constrains this desulfurization and oxidization of troilite to relatively low temperature. This low-temperature condition is consistent with the location of the Chang'e 6 landing region, which is at the edge of South Pole–Aitken basin.

Table S1.

Quantitative TEM-EDX result (atom%) of different positions on the Fe_2O_3 particle.

No.	Ti	Fe	O	Al	Si	S	Ca	Fe/O	Mineralogy
1	0.05	30.38	54.81	0.21	6.85	0.25	0.25	**0.55**	**Hematite**
2	0.25	31.48	53.11	0.24	7.01	0.06	0.32	**0.59**	
3	0.02	35.53	48.47	0.42	7.29	0.19	0.11	**0.73**	
4	0.04	29.66	53.28	0.34	8.46	0.18	0.22	**0.55**	
5	0.07	31.94	52.36	0.26	6.99	0.11	0.17	**0.61**	
6	0.11	34.79	50.33	0.33	6.22	0.19	0.20	**0.69**	
7	0.30	32.82	52.06	0.17	6.83	0.12	0.16	**0.63**	
8	0.10	31.01	55.87	0.19	4.38	0.14	0.20	**0.55**	
9	0.08	30.73	52.73	0.56	6.35	0.11	0.15	**0.58**	
10	0.11	32.62	51.49	0.56	4.61	0.59	0.26	**0.63**	
11	0.18	31.69	52.13	0.69	6.05	0.17	0.33	**0.60**	
12	0.23	33.23	50.58	0.33	6.66	0.31	0.21	**0.65**	
13	0.14	36.61	50.43	0.22	4.22	0.25	0.07	**0.72**	
14	0.21	34.87	50.68	0.48	4.07	0.27	0.28	**0.68**	
15	0.34	33.42	49.61	0.57	5.20	0.23	0.17	**0.67**	
16	nd	29.03	53.57	0.53	6.68	0.49	0.29	**0.54**	**Maghemite**
17	nd	29.41	51.58	0.34	6.82	1.61	0.29	**0.57**	
18	nd	28.08	54.86	0.35	6.79	0.17	0.16	**0.51**	
19	nd	30.58	50.85	0.41	6.07	1.32	0.12	**0.60**	
20	nd	26.73	52.81	0.17	8.99	0.66	0.00	**0.51**	
21	nd	28.80	50.93	0.34	6.55	0.60	0.18	**0.57**	
22	nd	30.51	51.97	0.21	7.29	0.22	0.08	**0.59**	
23	nd	30.15	51.26	0.19	6.94	0.59	0.32	**0.59**	

Note: nd = not detected.

Data S1. (separate file)

Raw data behind Figs. 1–4.
